# Antimicrobial and Anti-inflammatory Effects of a Novel Peptide From the Skin of Frog *Microhyla pulchra*

**DOI:** 10.3389/fphar.2021.783108

**Published:** 2021-12-16

**Authors:** Maolin Tian, Junfang Liu, Jinwei Chai, Jiena Wu, Xueqing Xu

**Affiliations:** ^1^ Department of Pulmonary and Critical Care Medicine, Zhujiang Hospital, Southern Medical University, Guangzhou, China; ^2^ Guangdong Provincial Key Laboratory of New Drug Screening, School of Pharmaceutical Sciences, Southern Medical University, Guangzhou, China

**Keywords:** antimicrobial peptide, *Microhyla pulchra*, amphibian, brevinin-2, LPS, anti-inflammation

## Abstract

Brevinins are an important antimicrobial peptide (AMP) family identified in the skin of *Ranidae* frogs and generally contain a characteristic ranabox structure at their C-terminal sequence. Herein a novel AMP named brevinin-2MP has been identified from the skin of the frog *Microhyla pulchra* by molecular cloning. Brevinin-2MP (GVITDTLKGVAKTVAAELLRKAHCKLTNSC) with a high amphipathic α-helix in sodium dodecyl sulfate solutions can destroy bacterial cell membrane and kill microbes. Furthermore, brevinin-2MP has been found to inhibit the lipopolysaccharide (LPS)-induced expression of pro-inflammatory NO, MCP-1, IL-6, and TNF-α *via* binding unidentified targets on the cell membrane and consequently suppressing the activation of MAPK/NF-κB signaling cascades induced by LPS in RAW 264.7 cells. Consistently, brevinin-2MP significantly alleviates the acute inflammatory response in carrageenan-induced mice paw. In conclusion, brevinin-2MP with anti-inflammatory and antimicrobial properties will be an ideal candidate drug molecule for bacterial inflammation treatment.

## Introduction

Antimicrobial peptides (AMPs) in amphibian skin which are encoded by ancient defensive genes play an important role in resisting microbial invasion and have evolved to various structures (Xu and Lai, 2015). Currently, according to structural similarity, AMPs found in amphibians have been divided into several peptide families, such as brevinin, esculentin, ranatuerin, cathelicidin, and so on. Brevinin-2 is a significant AMP superfamily with extensive structural characteristics and strong biological activities (Conlon et al., 2004). The first brevinin-2 was identified from the skin of the frog *Rana brevipoda porsa* (Morikawa et al., 1992), and more than 80 brevinin-2 peptides, including rugosin A and B, gaegurin 1–3, and nigrocin-1, have been subsequently isolated and identified from different Asian and European rather than North American Ranidae species up to now (Park et al., 1994; Park et al., 2001; Xu and Lai, 2015). It is well known that the primary structure of brevinin-2 peptides is greatly diverse in different species and even among different members belonging to the same species (Conlon et al., 2004; Conlon et al., 2007; Conlon et al., 2008a). In addition, it is apparent that brevinin-2 peptides can be divided two subfamilies with 29–30-residue and 33–34-residue peptides. However, almost all brevinin-2 peptides possess a net positive charge, a helical conformation, and a unique invariant disulfate loop structure called “ranabox”, consisting of the cyclic Cys-Lys-Xaa-Xaa-Xaa-Xaa-Cys at C-terminus (Conlon et al., 2009; Conlon et al., 2014; Savelyeva et al., 2014). Brevinin-2 peptides usually show strong antimicrobial activity against gram-negative *Escherichia coli* and gram-positive *Staphylococcus aureus* but a relatively low hemolyticity when compared to that of brevinin-1 peptides (Conlon et al., 2006; Conlon et al., 2007). Furthermore, brevinin-2GUb at 100 nM has been found to significantly promote insulin release (Conlon et al., 2008b). In addition, brevinin-2GUb (GVIIDTLKGAAKTVAAELLRKAHCKLTNSC) at a concentration of more 0.3199 μM has been reported to significantly reduce the release of TNF-α from ConA-stimulated peripheral blood mononuclear (PBM) cells and IFN-γ from unstimulated PBM cells, too (Popovic et al., 2012). However, no other activity is further reported from them so far.

The beautiful pygmy frog, *Microhyla pulchra*, with an average length of 33 mm in female and 30 mm in male individuals, respectively, widely inhabits south China (China Wildlife Protection Association, 1994). *M. pulchra* has been used as traditional Chinese medicine for therapy of surface inflammation and suppuration for several centuries (The Cooperative Group of Chinese Medicinal Fauna, 1983). However, no AMP has been reported from this species. In this article, a new AMP, brevinin-2MP (Bre-2MP in abbreviation), was identified from the skin of *M. pulchra*. In addition, we also found that brevinin-2MP had good anti-inflammatory activity *in vivo* and *in vitro*. It is the first AMP from *M. pulchra* and first report about brevinin-2 peptide with anti-inflammatory activity *in vivo*. The discovery will extend the function of brevinin-2, help understand the survival mechanism, and provide a candidate molecule for the development of new antibacterial and anti-inflammatory drugs.

## Materials and Methods

### Animals and Ethical Statement

Male and female adult *M. pulchra* frogs (*n* = 3), which are not protected or endangered species, were acquired in the countryside around Guangzhou City, Guangdong Province of China, and then were identified according to the species record website (https://amphibiaweb.org/) and the book “Atlas of Amphibians of China”. The mice used in the living animal experiment were bought from the experimental animal center of Southern Medical University (Guangdong, China). The mice were raised under standard acclimatization environments of 12-h light/dark cycle at 25°C and fed normal rodent feed procedure. All experimental processes involving living animals were permitted by the animal care and use ethics committee of Southern Medical University and implemented in strict accordance with the guidelines of the Committee for Animal Care and Use.

### Molecular Cloning and Characterization of cDNA Encoding Brevinin-2MP

The molecular cloning of cDNA encoding brevinin-2MP was carried out as reported previously by us with minor modification (Zeng et al., 2018). In short, the frogs were humanely euthanized with carbon dioxide, and their skins were stripped and cut into small pieces to extract total RNA using Trizol reagent (Life Technologies, Carlsbad, CA) in light of the manual of the manufacturer. The total cDNA in the skin was synthesized with the extracted RNA with a SMART™ cDNA Library Construction Kit (Takara biotechnology, Dalian, China) based on the protocol of the manufacturer and was used as the template for amplification of the cDNAs encoding brevinin-2MP with a combination of two oligonucleotide primers, HG (5′-AGA​TGA​AGA​AAT​CCC​TGT​TAC​TTC-3′) in the sense direction designed in light of the signal peptide of the reported precursor of brevinin-2GHa from *Hylarana guentheri* frog (Zhou et al., 2006) and CDS III (5′-ATT​CTA​GAG​GCC​GAG​GCG​GCC​GAC-3′) in the antisense direction in the PCR reaction. The PCR procedure was carried out in a mixture with Gene Tap polymerase (TianGen, Beijing, China), skin cDNA, and primers using a thermal cycler (Applied Biosystems, Foster, CA, United States). The PCR program was as follows: 4 min at 95°C; 30 cycles of 15 s at 94°C, 20 s at 46°C, and 20 s at 72°C; and, finally, at 72°C for 10 min for extension. The purified PCR product (about 250 bp) was cloned into pMD18-T vector (Takara biotechnology, Dalian, China) and sequenced by Applied Biosystems DNA analyzer, model 3730XL (Applied Biosystems, Foster, CA, United States).

### Bioinformatics Analysis

The chemical and physical parameters of the polypeptide were obtained *via* the Bioinformatics Resource Portal (http://www.expasy.org/tools/). The structure prediction of brevinin-2MP was performed *via* the trRosetta algorithm (https://yanglab.nankai.edu.cn/trRosetta/) with the NMR structure of gaegurin 4 (PDB entry 2G9L) as a template on the basis of maximum similarity (Yang et al., 2020). The comparative 3D structure model of brevinin-2MP was generated and optimized with Modeller 9.12 (University of California San Francisco, San Francisco, CA, United States). The generated 3D structural model was visualized using PyMOL software (Schrödinger) without any refinement. As further confirmation, the secondary structure of brevinin-2MP was further predicted with the Jpred4 and SOPMA secondary structure prediction servers provided by the Division of Computational Biology, School of Life Sciences, University of Dundee and PBIL-IBCP of the Institute of Biology and Protein Chemistry.

### Peptide Synthesis

Brevinin-2MP and brevinin-2MP labeled by FITC at the N terminal were synthesized by GL Biochem Ltd. (Shanghai, China). The crude brevinin-2MP peptide was purified by reversed-phase high-performance liquid chromatography on an inert silica gel ODS-SP column (Shimazu, Sumi, Japan) equilibrated with acetonitrile/water/trifluoroacetic acid at a flow rate of 1 ml/min. The peptide was eluted with 43% acetonitrile solution at 18 min. When the purity of the peptide, computed on the basis of the ratio of different front areas, was more than 95%, the peak was collected and lyophilized and further confirmed by MALDI-TOF mass spectrometry (Voyager-DE-PRO MALDI-TOF, AB SCIEX, Supplementary Figures S1, S2).

### Circular Dichroism Analysis

Circular dichroism (CD) was used to study the secondary structure of brevinin-2MP and to detect the changes of the secondary structure of brevinin-2MP in different solutions and temperatures. CD spectra were measured on Chirascan plus ACD spectropolarimeter (Applied Photophysics Ltd., Leatherhead, United Kingdom), which has a cell with 1 mm optical path length. The spectrum at 190–260 nm was determined at 25°C by a 0.1-cm-long cell with a width of 1 nm, scanning speed of 100 nm/min, and response time of 1 s. The samples were obtained by dissolving the brevinin-2MP powder in sodium dodecyl sulfate (SDS) solution (0, 30, 60, 90, and 120 mM) or 60 mM SDS solution containing a series of concentrations of sodium chloride (0, 100, 200, and 400 mM). For the temperature effect experiment, the peptide powder was dissolved in 60 mM SDS solution and incubated at different temperatures (25, 50, 70, and 90°C) for 1 h, and then the CD spectrum was measured. The concentration of peptide in all the above-mentioned samples was 100 μM. CD data are expressed as the mean residue ellipticity (*θ*) of three consecutive scans per sample in degree square centimeter per decimole. The following equation is used to calculate the mean residue ellipticity (*θ*, deg cm^2^ dmol^−1^):

$$\theta =\left(\theta_{\mathrm{obs}}\times \mathrm{1,000}\right)÷\left(c\times l\times n\right)$$

where θ_obs_ is the observed ellipticity (mdeg), *c* is the concentration (mM) of the peptide solution, *l* is the path length (mm), and *n* is the number of peptide residues.

### Antimicrobial Activities Analysis

The minimal inhibitory concentration (MIC) and the minimum bactericidal concentration (MBC) were measured to ensure the antimicrobial capacities of brevinin-2MP in 96-well plates, with a twofold dilution method as reported in our previous paper (Zeng et al., 2018). In brief, standard strains, including *E. coli* ATCC 25922, *Pseudomonas aeruginosa* ATCC 27853, *S. aureus* ATCC 25923, *Propionibacterium acnes* ATCC 6919, *Bacillus subtilis* CMCC 63501, and *Candida albicans* ATCC 10231, purchased from Guangdong Institute of Microbiology were grown to exponential phase at 37°C with Mueller–Hinton broth (MH broth) and diluted to 10^6^ CFUs/ml with fresh culture medium. Then, 50 μl/well of the resulting sample was then loaded to 96-well microplates which contained equal volumes of a serial dilution of brevinin-2MP with MH broth. After 16 h of gentle shaking while incubated at 37°C, the absorbance of the bacterial suspensions was read at 600 nm with a microplate reader (Tecan Trading AG, Männedorf, Switzerland). MIC was defined as the minimum concentration inhibiting visible growth. MBC was then determined following the MIC assay. Then, 10 μl of the sample which exhibited no evident growth after 16 h of incubation was inoculated onto MH agar plates. These plates were placed at 37°C for another 16 h. The MBC was defined as the peptide concentration at which there was no colony growth. In addition, the bacterial killing kinetics of brevinin-2MP against *E. coli* ATCC 25922 was carried out according to our previous method with minor modification (Zeng et al., 2018). Briefly, a final inoculum of 10^6^ CFUs/ml was grown in MH broth with brevinin-2MP (0×, 2×, and 4× MICs) at 37°C. Duplicate samples were withdrawn at 0, 15, 30, 60, 90, 120, 150, and 180 min before spreading on LB agar plates. Viable colonies were counted after the plates were placed for 16 h at 37°C. Ampicillin and sterile saline were used as the positive and negative controls, respectively. All experiments were repeated three times.

### Cytotoxicity and Hemolytic Assay

The cytotoxicity of brevinin-2MP on different mammal cells was examined by MTT. Three tumor cell lines (H460, M21, and MDA-MB-231) and two normal mammalian cells (RAW 264.7 macrophages and splenocytes) were inoculated in 96-well plates at a density of 5,000 cells per well and cultured in Dulbecco's modified Eagle’s medium (DMEM) or RPMI 1640 medium or in a medium containing continuous concentrations of brevinin-2MP (1.25, 2.5, 5, 10, and 20 μM) for 48 h at 37°C before the cell viabilities were measured with MTT methods as our reported methods (Chai et al., 2021). Hemolytic activity was also measured as described in our previous article with minor modification (Wu et al., 2021). In short, 2% of mouse erythrocyte suspension in Tris-buffered saline solution (v/v) was treated with different concentrations of brevinin-2MP (6.25, 12.5, 25, 50, and 100 μM) in a 96-V-well plate at room temperature for 2 h. Then, 1% Triton X-100 and phosphate-buffered saline (PBS) were applied as the positive and negative control, respectively. The absorbance of the supernatant at 540 nm was measured with a microplate spectrophotometer. The hemolysis ratio was counted with the following formula: percentage hemolysis = (OD_sample_ – OD_PBS_) / (OD_Triton_ – OD_PBS_) × 100%.

### Membrane Permeability and Morphology Change Observation

Both confocal laser scanning microscopy and scanning electron microscopy (SEM) experiments were performed to examine the potential antimicrobial mechanism of brevinin-2MP against the tested microorganisms according to our previous methods (Chai et al., 2021). In brief, *E. coli* and *S. aureus* at the late exponential phase were incubated with 1× MIC of brevinin-2MP at 37°C for 1.5 h. SYTO9 and propidium iodide (PI) from the LIVE/DEAD® BacLight kit (Invitrogen, Waltham, Massachusetts, United States) were incubated with the above-mentioned treated cells for 30 min at room temperature according to the instructions of the manufacturer. The stained cells were observed under confocal laser scanning microscopy (Leica TCS SP5; Leica Microsystems, Wetzlar, Germany) with emission/excitation wavelengths of 635/490 nm for PI and 500/480 nm for SYTO9, respectively. The living microbes with intact membrane were stained by SYTO9 emitting green fluorescence. However, the dead bacteria with broken membrane were stained by PI emitting red fluorescence. About 5–10 single-plane images per coverslip were obtained. For the SEM observation, bacterial suspensions at logarithmic growth phase (10^6^ CFUs/ml) were mixed with brevinin-2MP (2× MICs) for 30 min at 37°C. Then, the bacterial cells were harvested by centrifugation and sequentially fixed with 4% glutaraldehyde solution at room temperature for 4 h and 2.5% at 4°C overnight, respectively. After three times of washing with PBS, the bacteria were dehydrated sequentially with a series of concentration gradients of ethanol solution, followed by tert-butyl alcohol, and dried in a freeze-dryer (Quorum, UK). After gold coating, bacterial morphology was visualized by JSM-840 instrument (Hitachi, Japan) at a magnification of ×50,000; then, the bacterial morphology was captured at 5 kV voltage. A total of 5–10 single-plane images were obtained for each sample.

### Membrane Binding Assay

Membrane binding assays were undertaken with FITC-labeled brevinin-2MP according to our previous methods (Chai et al., 2021). In short, RAW 264.7 macrophages and bacterial cells in the logarithmic phase were prepared in PBS at a density of 1 × 10^5^ cells/ml and incubated with FITC-labeled brevinin-2MP (0, 2.5, 5, and 10 μM) for 15 min at 37°C. The unbound peptide was removed by washing with PBS containing 1% bovine serum albumin. Cell fluorescence intensity was detected with a FACscan flow cytometer (Becton Dickinson, United States). Cells without peptide treatment were regarded as the negative control.

### NO and Inflammatory Cytokine Generation Determination

RAW 264.7 macrophages were grown in DMEM medium with 10% FBS, 100 units per milliliter of ampicillin plus 100 μg/ml streptomycin, and seeded to 96-well plates at a density of 10^5^ cells per well. After cell adhesion, the cells were incubated with brevinin-2MP (0, 2.5, 5, and 10 μM) for 1 h before further incubation with LPS (100 ng/ml, *E. coli* O_55_: B_5_, L6529, Sigma Aldrich, St. Louis, Missouri, USA) in an incubator at 37°C for another 24 h. Then, the culture supernatants were collected to measure NO contents by Griess reagent (Beyotime Biotechnology, China), MCP-1, and IL-6 as well as TNF-α levels with enzyme-linked immunosorbent assay (Thermo Fisher Scientific, United States) in light of the manuals of the manufacturer. The cells without peptide and/or LPS incubation were considered as the negative control. Nitrite contents which reflect NO generation in medium were calculated from the standard curve obtained with NaNO_2_ (Sigma-Aldrich, St. Louis, Missouri, United States). All experiments were repeated three times.

### Western Blot Analysis

RAW 264.7 cells were seeded into six-well plates at 1 × 10^6^ cells per well and cultured in DMEM medium containing 10% FBS for 12 h. The cells were treated with different concentrations of brevinin-2MP (2.5, 5, and 10 μM) for 30 min and then stimulated with 100 ug/ml LPS for another 30 min. The cells lacking peptide and LPS treatment were considered as the negative control. All cells were collected by centrifugation and washed twice with pre-cooled PBS before the cytoplasmic or nuclear proteins were extracted using our previously published method for Western blot analysis (Nguyen et al., 2020). Primary antibodies against phospho-JNK/JNK, phosphor-ERK/ERK, phospho-p38/p38, NF-κB p65, Lamin A/C, and GAPDH (1: 1500; Cell Signaling Technology, Beverly, Massachusetts, United States) were applied in western blot analysis. All experiments were undertaken in triplicates.

### Paw Edema Assay

The anti-inflammatory function of brevinin-2MP *in vivo* was measured with the carrageenan-induced mouse paws as reported previously by us (Deng et al., 2021). Briefly, male and female Kunming mice (20–22 g) were randomly divided into four groups, with six mice in each group. Before the experiment, the paw volume up to the ankle joint was measured with a plethysmometer (Taimeng PV-200 7500, China) before injecting them with 50 μl normal saline containing 1% carrageenan into the plantar side of the paw. The mice were intraperitoneally injected with brevinin-2MP (10 mg/kg), saline, or indomethacin (5 mg/kg) for 1 h. The paw volume up to the ankle joint was surveyed at 1, 2, 4, 8, and 24 h after the carrageenan administration. The increasing volume was calculated by the delta volume ( a−b ), where “a” and “b” are the volumes of the right hind paw after and before carrageenan administration, respectively. In the other experiments, the secondary batch of mice was injected sequentially with brevinin-2MP, indomethacin, saline, and carrageenan as mentioned above. The right hind paws of all mice were surgically cut at 4 h post-injection for histological examination and myeloperoxidase (MPO) activity analysis.

### Data Analysis

The statistical analysis of data was performed with Igor and GraphPad Prism 5.0 software (GraphPad Software Inc., La Jolla, CA, United States). One-way analysis of variance with Bonferroni’s multiple-Comparison test was carried out to judge the significance when comparing two or more groups with a control group and with a *post-hoc* Tukey test when making comparisons among three or more groups. The unpaired Student’s *t*-test was implemented to measure the significance between two experimental groups. Data are shown as mean ± SEM.

## Results

### Identification and Characterization of Brevinin-2MP

**FIGURE 1:**
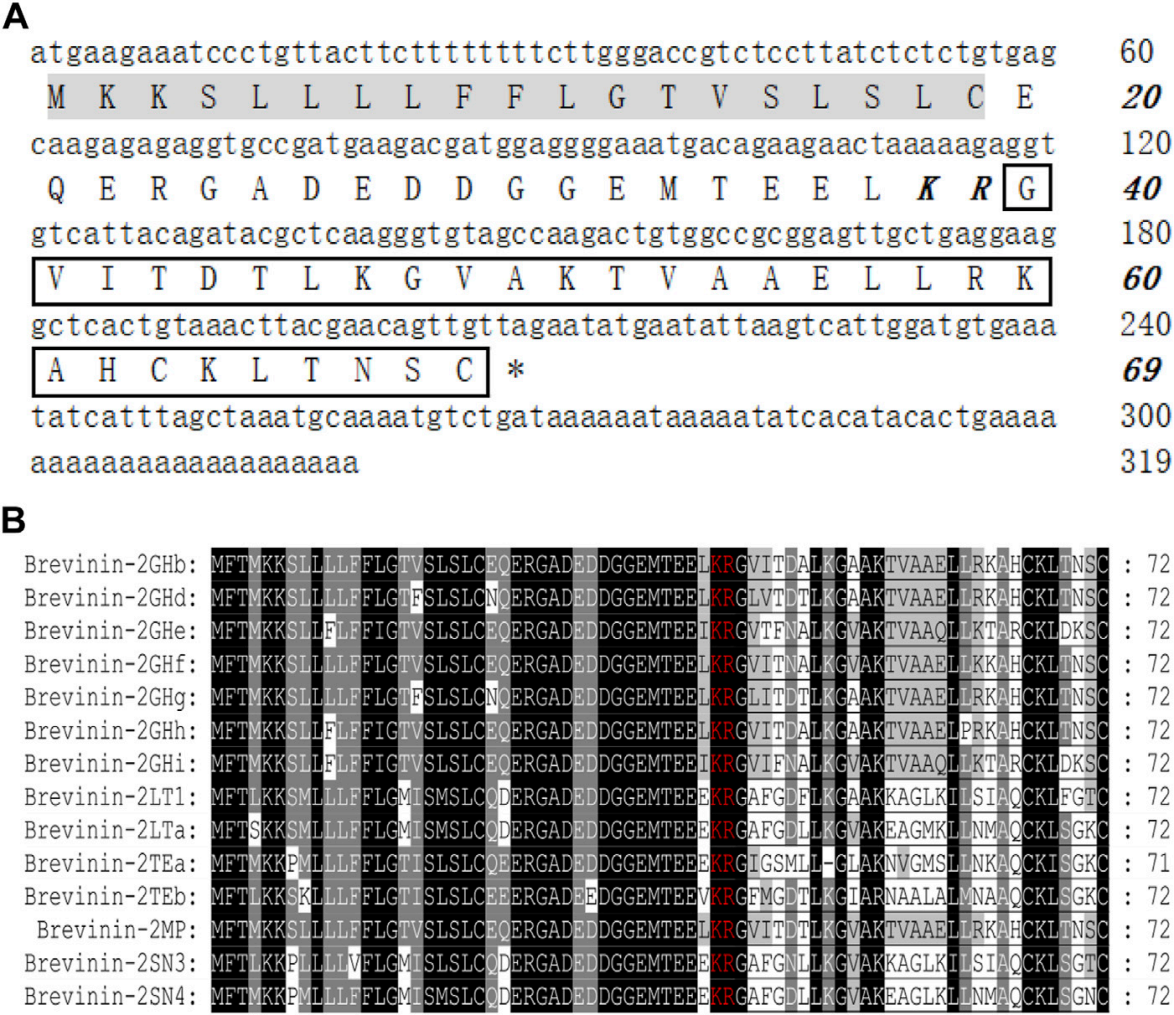
Sequence characterization of brevinin-2MP. **(** **A** **)** The cDNA and deduced amino acid sequences of brevinin-2MP. The signal peptide is marked with a gray background, and the KR residues in italic bold type indicate the end of an acidic spacer domain. The sequence of mature peptide is boxed, and the stop codon is denoted by an asterisk (*). **(** **B** **)** Multi-sequence alignment of the brevinin-2 precursors from frogs. The KR residues at the end of the acid spacer domain are marked in red, and the same amino acid residues are represented with a black background.

The cDNA sequence encoding AMP was obtained from *M. pulchra* frog skin gene library by PCR-based cloning technique. As displayed in Figure 1A, the predicted protein precursor consisted of 69 amino acid residues containing a 19-residue signal peptide, an acidic domain with 20 residues, and a mature peptide with 30 residues following the KR residues which form the classic protease cleavage site of frog defense peptide. NCBI Basic Local Alignment Search Tool (BLAST) analysis showed that the predicted precursor sequence highly resembled those of brevinin-2 peptides. Especially, they shared complete identity in the signal peptide and acidic domains of brevinin-2 peptides from *Sylvirana guentheri* . In addition, the sequence of mature peptide (GVITDTLKGVAKTVAAELLRKAHCKLTNSC) had only several residue differences with brevinin-2 peptides from *S. guentheri* ; for example, the former was only two amino acid different from brevinin-2GHb and brevinin-2GUb (Figure 1B) (Conlon et al., 2008a; Dong et al., 2017). Hence, we judged that this new peptide belonged to the brevinin-2 family and named it as brevinin-2MP. Brevinin-2MP possessed a theoretical PI of 9.31 with +3.01 net charge and aliphatic index of 107.33. The grand average of hydropathicity (GRAVY) of brevinin-2MP was predicted to be 0.210. Its relative mass without the intramolecular disulfide bond was predicted to be 3,141.71, which was nearly identical with the value shown in Supplementary Figure S2A from the mass spectrum result.

### Secondary Structure of Brevinin-2MP

**FIGURE 2:**
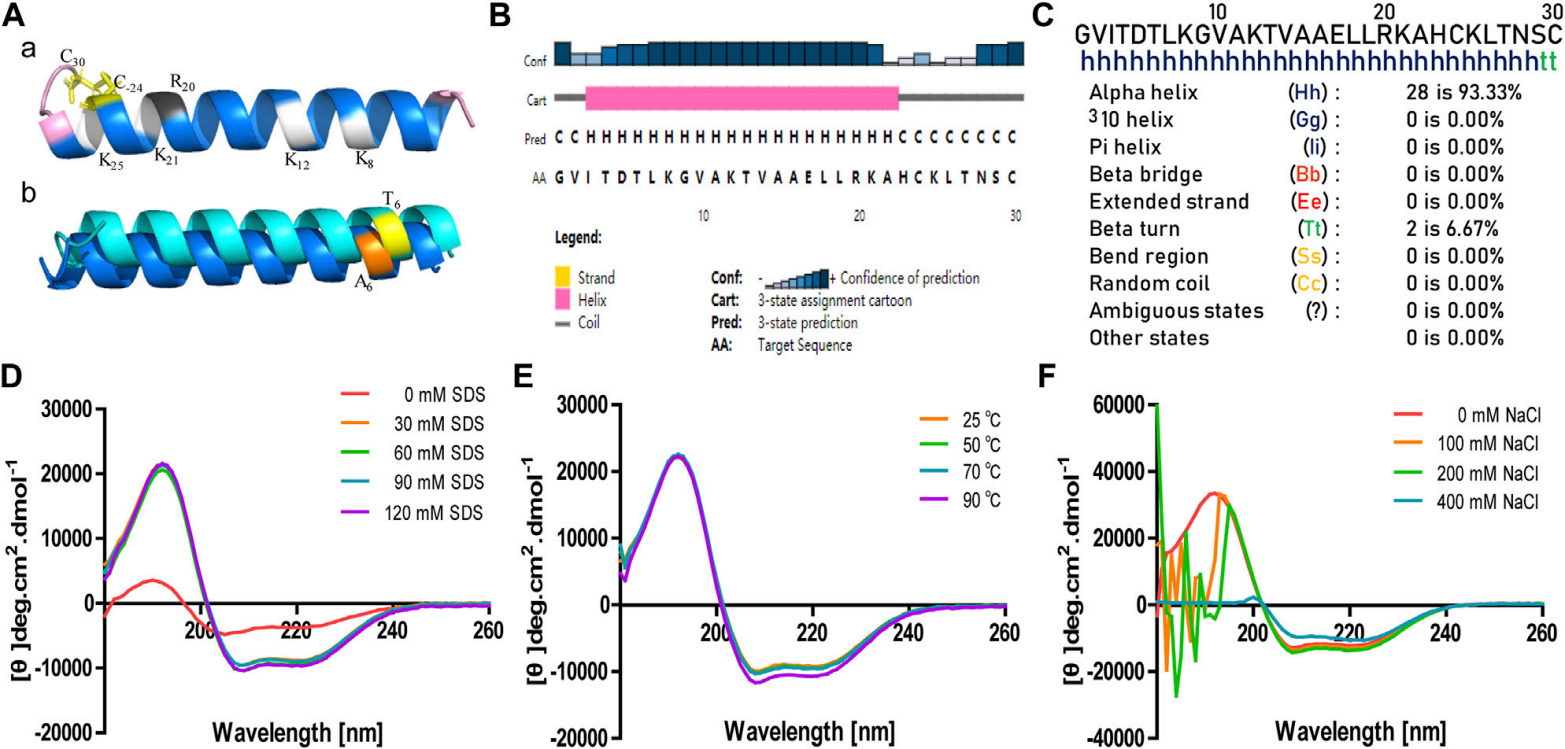
The predicted secondary structure and CD analysis of brevinin-2MP. **(** **A** **)** Secondary structure modeling of brevinin-2MP produced by trRosetta (a) and secondary structure comparison of brevinin-2MP and brevinin-2GHb (b) visualized with PyMOL. The predicted brevinin-2MP structure is shown in the form of ribbons, with sky blue indicating α-helix and pink indicating irregular coils, and the different amino acids of brevinin-2MP and brevinin-2GHb are labeled with different colors. **(** **B**, **C** **)** Secondary structures of brevinin-2MP predicted by the Jpred4 and SOPMA secondary structure prediction servers provided by the Division of Computational Biology, School of Life Sciences, University of Dundee and PBIL-IBCP of the Institute of Biology and Protein Chemistry. **(** **D**–**F** **)** The CD spectra of brevinin-2MP in the indicated conditions.

The secondary structure of brevinin-2MP was predicted with gaegurin 4 (PDB entry 2G9L) as the homologous template because the modeling credibility is very high, with a confidence of 96.3 and E-value of 0.00047 according to trRosetta algorithm search (Yang et al., 2020). Figure 2A suggests that the α-helix was the main conformation component in the predicted brevinin-2MP structure. In addition, residues G1-I3 and N28-C30 formed one coil at the N-terminal part and at the C-terminal part of the structure, respectively (Figure 2A). Furthermore, the major secondary structural components of brevinin-2MP predicted by both Jpred4 and SOPMA were also α-helix, which took 80.00 and 93.33% of the whole structure in the latter two methods, respectively (Figures 2B,C). In order to verify the prediction accuracy, the CD spectra of brevinin-2MP in different solutions were measured. As described in Figure 2D, the CD spectra of brevinin-2MP dissolved in H_2_O presented a large negative peak at 205 nm and a small negative peak at 223 nm, which is the spectral characteristic of random coil. Subsequently, one positive peak at 195 nm and two negative peaks at 208 and 222 nm appeared in the CD spectra of brevinin-2MP dissolved in SDS solution, which suggested that the secondary conformation of brevinin-2MP was mainly composed of α-helix. The α-helix structure accounts for 96.42% of the mature peptide chain of brevinin-2MP, according to CDNN program calculation. In addition, the secondary structure of brevinin-2MP was hardly affected by SDS at the concentration range of 30–120 mM (Figure 2D). Similarly, the CD spectra of brevinin-2MP showed no significant difference after treatment at different temperatures, even at 90°C (Figure 2E). However, despite the fact that two negative peaks at 208 and 222 nm still existed with the increasing concentrations of sodium chloride in SDS solution, the positive peak at 195 nm disappeared, and some other peak appeared, which was mostly due to the severe signal distortion known to happen at high chloride concentrations (Figure 2F).

### Antimicrobial Activities Assay

The antimicrobial properties of brevinin-2MP against Gram-negative and Gram-positive bacteria as well as fungi are shown in Table 1. Brevinin-2MP possessed antimicrobial activity against tested strains other than *Pseudomonas aeruginosa*. Among them, brevinin-2MP had the strongest activity against *Bacillus subtilis* CMCC 63501, with a MIC value of about 4.97 μM. What is more, the killing kinetics of brevinin-2MP against *E. coli* ATCC 25922 was studied by colony counting assay. At concentrations of 2× MICs, brevinin-2MP was able to completely eliminate all the microbes in 120 min, and *E. coli* ATCC 25922 could not restart growth on agar plates after treatment, which suggested that brevinin-2MP is bactericidal rather than bacteriostatic (Supplementary Table S1).

**TABLE 1:** Antimicrobial activity of brevinin-2MP.

Microorganism	MIC/MBC (μM)	
	Brevinin-2MP	AMP
Gram-negative bacteria		
*Escherichia coli* ATCC 25922	47.78/47.78	100/100
*Pseudomonas aeruginosa* ATCC 27853	>100/>100	>100/>100
Gram-positive bacteria		
*Staphylococcus aureus* ATCC 25923	47.78/47.78	100/100
*Propionibacterium acnes* ATCC 6919	14.93/14.93	25/50
*Bacillus subtilis* CMCC 63501	4.97/4.97	25/50
Fungi		
*Candida albicans* ATCC 10231	59.73/59.73	50/100

### Effects on Bacterial Cell Membrane

**FIGURE 3:**
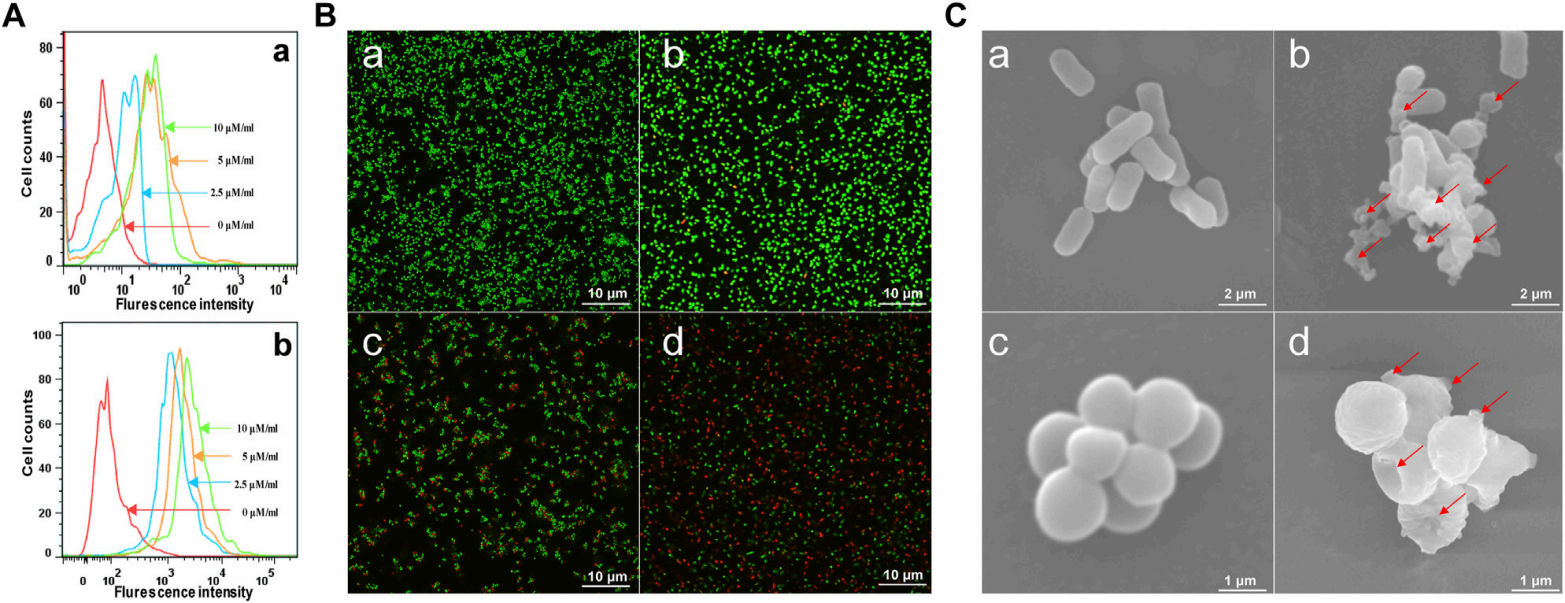
Antimicrobial mechanism of brevinin-2MP. **(** **A** **)** Flow cytometry analysis of the interaction between FITC-labeled brevinin-2MP and *S. aureus* ATCC 25923 (a) as well as *E. coli* ATCC 25922 (b). **(** **B** **)** Confocal laser scanning microscopy. The bacteria treated with or without brevinin-2MP were stained with LIVE/DEAD BacLight^TM^ Microbial Viability kits before observation by confocal fluorescence microscopy. *S. aureus* ATCC 25923 (a) and *E. coli* ATCC 25922 (b) without drug treatment; (c and d) *S. aureus* ATCC 25923 and *E. coli* ATCC 25922 after 1 h of treatment with brevinin-2MP. **(** **C** **)** The morphology of bacteria under a scanning electron microscope. *E. coli* ATCC 25922 (a) and *S. aureus* ATCC 25923 (c) without drug treatment. *E. coli* ATCC 25922 (b) and *S. aureus* ATCC 25923 (d) after 1 h of treatment with brevinin-2MP.

The attraction and attachment of AMPs to microbial cell surfaces are crucial to exert their antimicrobial activity (Vineeth and Sanil, 2017). Flow cytometry experiments were implemented to explore the binding of brevinin-2MP to bacteria. As described in Figure 3A, compared with the untreated group, the bacterial fluorescence intensities were enhanced in a concentration-dependent manner after *E. coli* ATCC 25922 and *S. aureus* ATCC 25923 were incubated with FITC-labeled brevinin-2MP for 15 min, indicating that brevinin-2MP could bind to them. It is well known that most AMPs exert their antibacterial activity by penetrating and destroying the integrity of the cell membrane. In order to determine the antibacterial mechanism of brevinin-2MP, both laser confocal scanning microscopy and scanning electron microscopy were applied. As shown in Figure 3B, only individual *E. coli* ATCC 25922 and *S. aureus* ATCC 25923 in the untreated control group were dyed red by PI, while most of the living bacteria were dyed green by STYO9. However, after treatment with brevinin-2MP of 1× MIC for 1 h, a large number of bacteria were dyed red, and the intensity ratio of PI/STYO9 was significantly increased, which indicated that brevinin-2MP, like most AMPs, obviously destroyed the integrity of the cell membrane and induced the inflow of PI into Gram-positive and Gram-negative bacteria. Consistently, the scanning electron microscopy images suggested that brevinin-2MP could damage the bacterial membrane. In comparison with the control group, the bacterial cell membrane treated with brevinin-2MP had an obvious expansion, deformation, and even a large amount of intracellular inclusion overflow (Figure 3C). All these results suggested that the cellular membrane permeabilization and disruption mechanism were responsible for the antimicrobial activity of brevinin-2MP.

### Cytotoxicity of Brevinin-2MP

As shown in Table 2, brevinin-2MP was a low hemolytic against mouse erythrocyte, and the hemolysis ratio was only 1.42 ± 0.16% even at the highest concentration of 100 uM. The effects of brevinin-2MP on the proliferation of splenocytes, RAW 264.7, H460, M21, and MDA-MB-231 cells were measured by the MTT assay. As reported in Table 3, H460 is the most sensitive to brevinin-2MP with a IC_50_ value of about 5.77 ± 1.21 μM after 48 h of exposure. However, brevinin-2MP showed low cytotoxicity to other tested mammalian cell lines, with IC_50_ values of more than 25 μM (Table 3).

**TABLE 2:** Hemolytic activity of brevinin-2MP.

Brevinin-2MP (μM)	Hemolysis ratio (%)
100	1.42 ± 0.16
50	0.78 ± 0.07
25	0.63 ± 0.16
12.5	0.39 ± 0.17
6.25	0.10 ± 0.20

**TABLE 3:** Cytotoxicity of brevinin-2MP.

Cells	IC_50_ (μM)
RAW 264.7	72.53 ± 2.16
Mouse splenocytes	60.32 ± 5.67
MDA-MB-231	26.36 ± 4.91
H460	5.77 ± 1.21
M21	60.41 ± 8.78

The results represent mean ± SEM, values from three separate experiments.

RAW 264.7, mouse leukemic monocyte macrophage cells; H460, human lung adenocarcinoma cells; MDA-MB-231, human breast cancer cells; M21, human melanoma cells;IC50; half-maximum inhibitory concentration.

### Inhibition of Inflammatory Factor Production Induced by LPS

**FIGURE 4:**
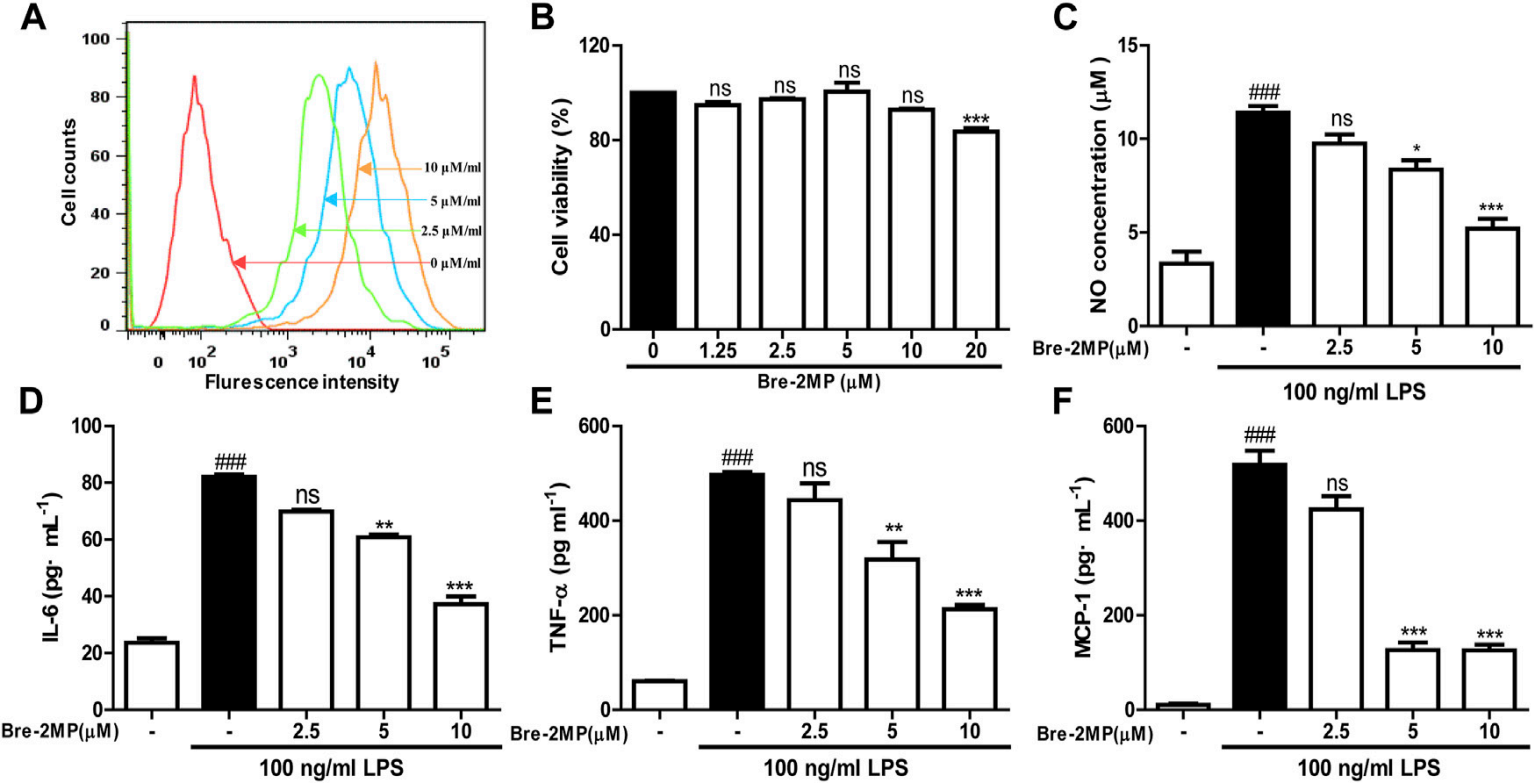
Effects of brevinin-2MP on lipopolysaccharide (LPS)-induced production of nitrite and cytokines. **(** **A** **)** The binding of brevinin-2MP to RAW 264.7 cells. FITC-labeled brevinin-2MP (2.5, 5, and 10 μM) were incubated with RAW 264.7 cells for 15 min at 37°C before flow cytometry analysis was carried out. **(** **B** **)** Effect of brevinin-2MP on the viability of RAW 264.7 cells. **(** **C**–**F** **)** Effect of brevinin-2MP on the production of NO, IL-6, TNF-α, and MCP-1 in RAW 264.7 cells induced by LPS. Data are mean ± SEM (*n* = 4). ∗*p* < 0.05, ∗∗*p* < 0.01 and *p* < 0.001 indicate significant difference compared with the control that was incubated with 100 ng/mL LPS. ^###^ *p* < 0.001 indicates significant difference in comparison with the control without LPS and brevinin-2MP.

The binding of AMPs to their targets on the surface of macrophages sets off cellular signaling pathway and regulates the secretion of pro-inflammatory factors. Therefore, the binding of brevinin-2MP to RAW 264.7 cells was firstly evaluated with flow cytometry. As shown in Figure 4A, similar with its binding with bacteria, after the FITC-labeled brevinin-2MP and RAW 264.7 cells were co-incubated for 15 min, obvious binding phenomenon occurred, and their binding effects were enhanced with the increased concentrations of brevinin-2MP. To define whether brevinin-2MP can affect the release of inflammatory cytokines in RAW 264.7 macrophages stimulated by LPS, we tested secondly the effect of brevinin-2MP on the viability of RAW 264.7 cells. As shown in Figure 4B, brevinin-2MP at the tested concentrations had no cytotoxicity toward RAW 264.7 cells. Thirdly, the contents of NO, TNF-α, and IL-6 in RAW 264.7 cells were investigated as illustrated in Figures 4C–F. Compared with RAW 264.7 cells without LPS stimulation, RAW 264.7 cells stimulated with 100 ng/ml LPS significantly increased the release of NO, MCP-1, IL-6, and TNF-α, while their increase trends were inhibited by pre-incubation with brevinin-2MP for 1 h before LPS stimulation. The inhibition rates of 2.5, 5, and 10 μM brevinin-2MP on these factors increased in RAW 264.7 cells to about 14–54%, 18–75%, 15–54%, and 10–57%, respectively.

### Inhibition of Inflammatory Response Pathways Activated by LPS

**FIGURE 5:**
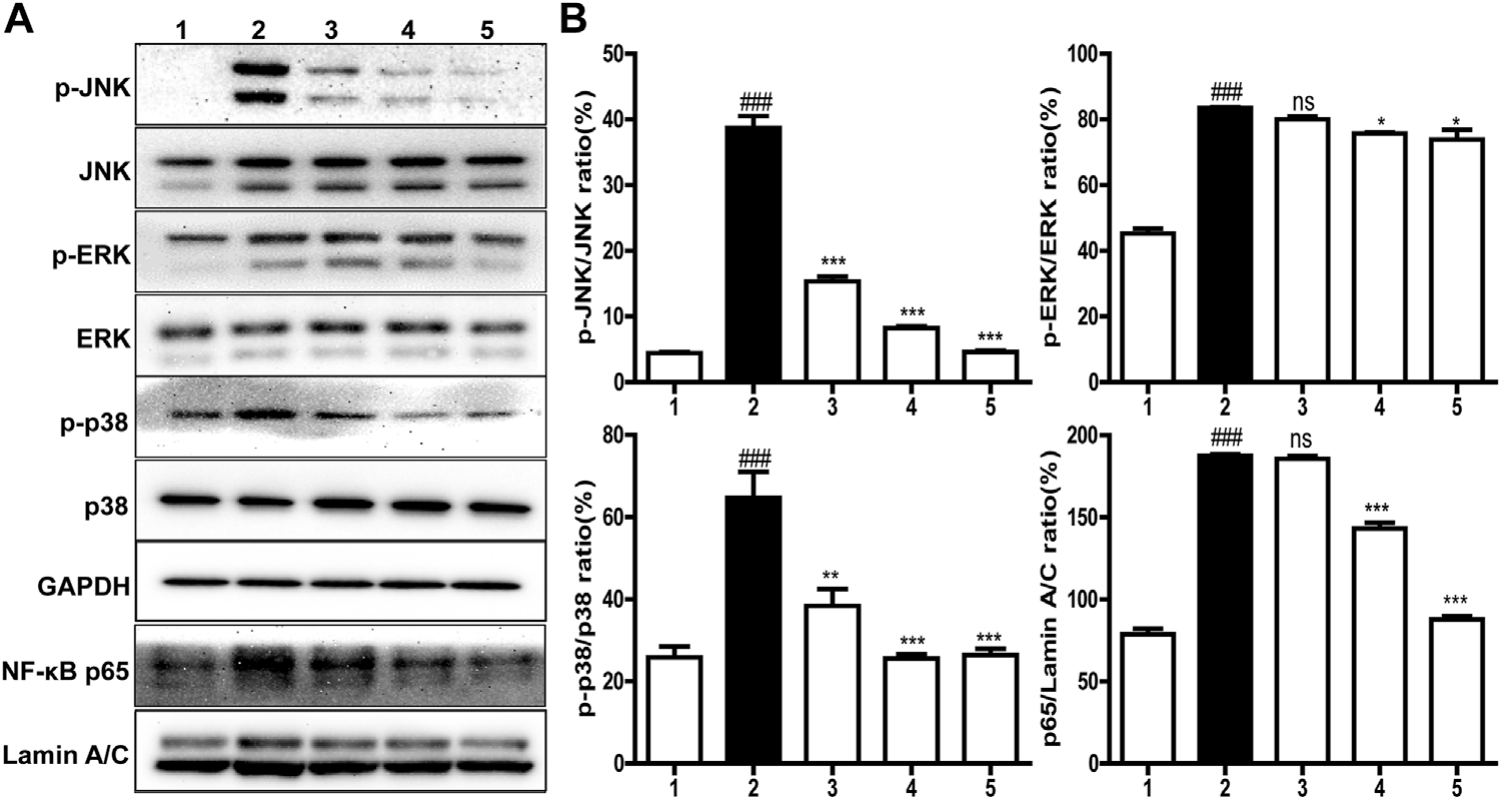
Effects of brevinin-2MP on lipopolysaccharide (LPS)-induced inflammatory pathways. **(** **A** **)** Typical western blot images of ERK, JNK, p38, and p65 in RAW 264.7 cells. The cells were incubated with 100 ng/ml LPS and 2.5, 5, and 10 μM brevinin-2MP for 30 min and then were harvested for western blot examination lines 1–5 are the control, LPS-treated cells, the cells treated by 100 ng/ml LPS plus 2.5, 5, and 10 μM brevinin-2MP, respectively. **(** **B** **)** Statistical analysis of band densities in western blot images. The results are shown as ratios of phosphorylated proteins to the corresponding total ones and p65 to Lamin A/C. Band densities were quantified using Quantity One software (Bio-Rad, Richmond, CA, United States). Data are presented as mean ± SEM (*n* = 3). ∗*p* < 0.05, ∗∗*p* < 0.01, and ∗∗∗*p* < 0.001 represent significant difference in comparison with the control treated only with LPS. ^###^ *p* < 0.001 represents significant difference with the control without brevinin-2MP and LPS.

It is well known that inflammatory response is closely related to the MARK/NF-κB signaling pathways (Akira et al., 2006). Thus, the expression of MARK/NF-κB signal proteins, like p38, JNK, ERK, and p65, was examined by western blot. As shown in Figures 5A,B, the contents of the p65 subunit translocated into the nucleus and phosphorylated p38 and ERK as well as JNK in 100 ng/ml LPS induced cells were significantly higher than those in normal cells, but these upregulations were inhibited in a concentration-dependent manner by brevinin-2MP which did not change the total protein levels of ERK, JNK, and p38. Specifically, after the administration of 10 μM brevinin-2MP, the contents of phosphorylated JNK, ERK, p38, and p65 in the nucleus were reduced by 11.54, 59.17, 88.14, and 53.26%, respectively. These results suggested that brevinin-2MP exerted its anti-inflammatory effect by inactivating the MAPK/NF-κB pathways in LPS-sensitized macrophage cells.

### Anti-inflammatory Effects on Carrageenan-Induced Paw

**FIGURE 6:**
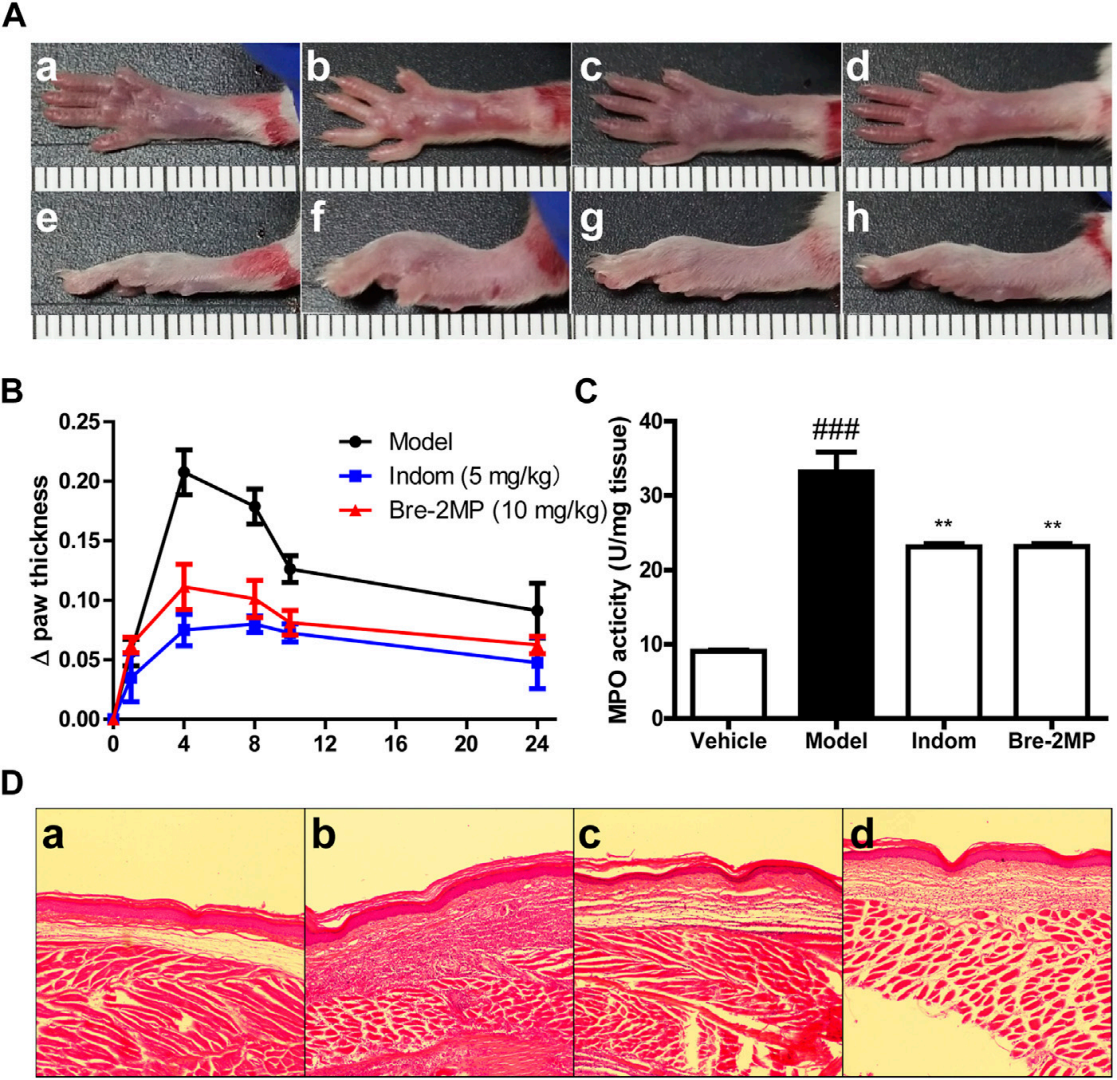
Inhibition of inflammatory response by brevinin-2MP in carrageenan-induced paw. A total of 50 μl of 1% carrageenan in the absence or presence of 10 mg/kg brevinin-2MP and 5 mg/kg indomethacin was injected into the mice paws before paw swelling; myeloperoxidase (MPO) activities and histological measurement were performed as well. **(** **A** **)** Mice paw photographs at 4 h post-injection. The labels a–d and e–h refer to the bottom and lateral views of the paws, respectively. a and e: paws from saline control group; b and f: paws from carrageenan plus saline group; c and g: paws from carrageenan plus indomethacin group; d and h: paws from carrageenan plus brevinin-2MP group. The scale unit shown in the images is centimeter. **(** **B** **)** Paw swelling volume at different time points after carrageenan administration. **(** **C** **)** MPO activity in the paws. Columns from left to right represent the MPO activity in the mice paws from the groups treated with saline, carrageenan plus saline, carrageenan plus indomethacin, and carrageenan plus brevinin-2MP, respectively. **(D)** HE staining of the mice paws at 4 h post-injection. The labels a–d refer to images from mice paws treated with saline, carrageenan plus saline, carrageenan plus indomethacin, and carrageenan plus brevinin-2MP, respectively. The scale bars were 50 μm. The statistical results are mean ± SEM (*n* = 6). **p < 0.01: significant difference in comparison with the control uniquely injected with carrageenan. ^###^ *p* < 0.001: significant difference with the control without the injection of brevinin-2MP and carrageenan.

The effect of brevinin-2MP on acute inflammation was evaluated by a carrageenan-stimulated paw edema assay. As shown in Figures 6A,B, the swelling degree of the paw reached a maximum at 4 h after the carrageenan injection into the right paw. Compared with the model group, the swelling rates of the right paw injected with indomethacin and brevinin-2MP at 4 h were decreased by about 63.85 and 46.38%, respectively. At 24 h after injection, their swelling rates were reduced by 47.94 and 31.50%, respectively. The increasing MPO activity indicates neutrophil infiltration. As shown in Figure 6C, the MPO activity was 9.09 ± 0.19 units/mg protein in the control group, while it was significantly increased by carrageenan administration and reached 33.16 ± 3.77 units/mg protein in the model group only injected with carrageenan. This increase induced by carrageenan was obviously suppressed by indomethacin and brevinin-2MP, and the MPO activity in their treatment groups was 23.13 ± 0.53 and 23.15 ± 0.50 units/mg protein, respectively. Coincidentally, the histological results showed that subcutaneous edema and inflammatory cell infiltration appeared in all carrageenan-injected paws, while these were less intense in the groups injected with indomethacin or brevinin-2MP when compared to the group injected only by carrageenan (Figure 6D).

## Discussion and Conclusion

A large number of AMPs belonging to brevinin-2 family have been widely identified from amphibian skin in the past few decades. Despite the fact that *M. pulchra* has been widely used as traditional Chinese medicine to treat surface inflammation and suppuration in south China for several centuries, no AMP has been reported currently from this species. Additionally, as far as we know, although some brevinin-2 peptides have been reported to contain antimicrobial, insulin-releasing, and cytotoxic activities as well cytokine suppression effects in PBM cells (Conlon et al., 2008b; Popovic et al., 2012; Chen et al., 2020; Zhong et al., 2020), none of them has been found to have anti-inflammatory activity in macrophage cells and in *in vivo* models. In this study, a novel AMP named brevinin-2MP with antimicrobial and anti-inflammatory activity has been identified from the skin of the frog *M. pulchra* by molecular cloning.

Most of the AMPs identified from the frog so far adopt an amphipathic α-helical conformation in a membrane environment stimulated by SDS solutions (Powers and Hancock, 2003). The structure prediction and CD analysis confirm that the secondary structure of brevinin-2MP is mainly the α-helix in SDS solution (Figure 2), indicating that brevinin-2MP can fold immediately into α-helix conformation after attaching to the bacterial outer membrane. As shown in Table 1, brevinin-2MP shows antimicrobial activity against Gram-negative and Gram-positive bacteria but very weak activity against *P. aeruginosa* ATCC 27853. Interestingly, compared with brevinin-2GHb with only two different amino acids, brevinin-2MP inhibits the growth of *E. coli* ATCC 25922 (MIC = 47.78 μM) less effectively than brevinin-2GHb (MIC = 2.7 μM), according to MIC values reported by Jianwu Zhou (Zhou et al., 2006). Therefore, the different primary sequence is responsible for their variation in antimicrobial activity against *E. coli*. The primary sequences of AMPs have greatly affected their net charges, size, and hydrophobicity, which are regarded as the primary factors to decide their antimicrobial activity (Nguyen et al., 2011). Considering that both brevinin-2MP and brevinin-2GHb have the same isoelectric point value of 9.31, we rule out that these differences in antibacterial activity are due to the effect of cationic degree on the antibacterial activity of brevinin-2MP. The Thr6 of brecvinin-2MP is a polar amino acid, while the Ala of brevinin-2GHb at the same position is a non-polar amino acid, which makes its hydrophobicity higher than that of brevinin-2MP (Figure 2A, panel b). Therefore, we judge that the different antimicrobial activity between two highly similar brevinin-2 peptides may result from the amino acid substitution decreasing the hydrophobicity, which results in changed bacterial membrane permeabilization (Chen et al., 2007). The increasing evidences have proved that most AMPs adopt an α-helical conformation target to the cell membranes and insert themselves into lipid bilayers, resulting in the formation of membrane pores (Gopal et al., 2012; Koehbach and Craik, 2019). Consistently, after binding with bacteria, brevinin-2MP can destroy the cell membrane, causing the outflow of bacterial contents and, finally, their death (Figure 3). Above all, the present study provides a reference for the structural optimization of AMPs discovered from amphibian species.

Lipopolysaccharide, an endotoxin component of the outer cell wall of Gram-negative bacteria (Maldonado et al., 2016), can bind to toll-like receptor 4 (TLR4), then activate transcription factor NF-κB, and promote the secretion of inflammatory factors like IL-6 and TNF-α, eventually leading to an inflammatory response (Rossol et al., 2011; Plociennikowska et al., 2015). It has been reported that many AMPs can inhibit the inflammatory response *in vitro* and *in vivo*. For instance, LL-37 and indolicidin have been reported to suppress TNF-α production from THP-1 cells stimulated by LPS (Gough et al., 1996; Bowdish et al., 2005). Similarly, esculentin-1GN and cathelicidin-MH can block the ability of LPS to induce inflammatory factors in RAW 264.7 macrophages and in carrageenan-induced mice paw plus LPS- and cecal ligation and perforation-induced septicemic mice (Zeng et al., 2018; Chai et al., 2021). In agreement, brevinin-2MP can evidently suppress the production of pro-inflammatory NO, TNF-α, IL-6, and MCP-1 in LPS-induced RAW 264.7 cells *via* suppressing the activation of the MAPK/NF-κB pathway induced by LPS (Figures 4, 5), which is further confirmed by a carrageenan-induced paw model *in vivo* (Figure 6). CAP37-derived peptides and Hc-CATH have been reported to bind TLR4. It seems that esculentin-1GN and cathelicidin-MH can also bind an unidentified target on the surface of macrophages. Considerably, brevinin-2MP can bind to RAW 264.7 cells rather than LPS (Figure 4A) and reduce carrageenan-stimulated inflammation in mice paw in the absence of LPS (Figure 6). In addition, brevinin-2GUb, which has a two-residue difference from brevinin-2MP, can suppress the secretion of TNF-α from Con A-induced PBM cells and IFN-γ from unstimulated PBM cells (Popovic et al., 2012). Thus, it is reasonable to speculate that brevinin-2MP should target the unidentified receptor on RAW 264.7 cells, which may also be expressed on the PBM cells, leading to the inactivation of the downstream signaling pathway and ultimately modulation of the production of inflammatory mediators.

In conclusion, this study reports a new antimicrobial and anti-inflammatory brevinin-2 peptide, brevinin-2MP. Brevinin-2MP can bind bacteria and show a significant antimicrobial activity against a range of bacteria by destroying the bacterial cell membrane. Meanwhile, brevinin-2MP can significantly regulate the secretion of NO, TNF-α, IL-6, and MCP-1 in LPS-induced RAW 264.7 cells by blocking the MARK/NF-κB inflammatory pathways. In addition, the mice paw edema assay further proves the anti-inflammatory capability of brevinin-2MP *in vivo*. Therefore, brevinin-2MP will be a new therapeutic drug with both antimicrobial and anti-inflammatory activities.

## Data Availability

The original contributions presented in the study are included in the article/Supplementary Material. Further inquiries can be directed to the corresponding author.

## Supplementary Material

Supplementary Figure S1 RP**-**HPLC analysis of brevinin-2MP.

Supplementary Figure S2 MALDI-TOF-MS analysis of brevinin-2MP without the intramolecular disulfide bond **(A)** and with the intramolecular disulfide bond **(B)**.
