# Supplementary material for: Antimicrobial and Anti-inflammatory Effects of a Novel Peptide From the Skin of Frog Microhyla pulchra
Source: Front Pharmacol. 2021 Dec 16;12:783108. doi: 10.3389/fphar.2021.783108 (PMC8718063; doi:10.3389/fphar.2021.783108)
Supplement: Supplementary file 1 [file Table1.DOCX]

| **Supplementary Table 1. The bacterial killing kinetics of brevinin-2MP against *E. Coli* ATCC 25922** | | | | | | | |
| --- | --- | --- | --- | --- | --- | --- | --- |
| **Time** | **Colony Forming Units (×10^5^, CFUs/ml)** | | | | | | |
|  | **0** | **30 min** | **60 min** | **90 min** | **120 min** | **150 min** | **180 min** |
| 0×MIC | 46 ± 4.00 | 64.5 ± 1.5 | 77 ± 2.00 | 77 ± 1.00 | 79 ± 1.00 | 185 ± 55.00 | 231 ± 30.00 |
| 2×MICs | 41 ± 1.00 | 21 ± 1.00 | 6.5 ± 1.50 | 1.5 ± 0.50 | 0 | 0 | 0 |
| 4×MICs | 28 ± 2.00 | 7 ± 2.00 | 2.5 ± 0.50 | 0.5 ± 0.50 | 0 | 0 | 0 |
| *E. coli* ATCC 25922 was mixed with brevinin-2MP at a concentration of 2×, 4× MICs for 0, 30, 60, 90, 120, 150 and 180 min, respectively. The MIC of brevinin-2MP against *E. coli* ATCC 25922 is 47.78 µM. | | | | | | | |

Supplementary Material

**Supplementary Figure 1.** RP**-**HPLC analysis of Brevinin-2MP.

**Supplementary Figure 2.** MALDI-TOF-MS analysis of Brevinin-2MP without the intramolecular disulfide bond (A) and with the intramolecular disulfide bond (B).
