# Supplementary figures and images for: Antimicrobial and Anti-inflammatory Effects of a Novel Peptide From the Skin of Frog Microhyla pulchra
Source: Front Pharmacol. 2021 Dec 16;12:783108. doi: 10.3389/fphar.2021.783108 (PMC8718063; doi:10.3389/fphar.2021.783108)

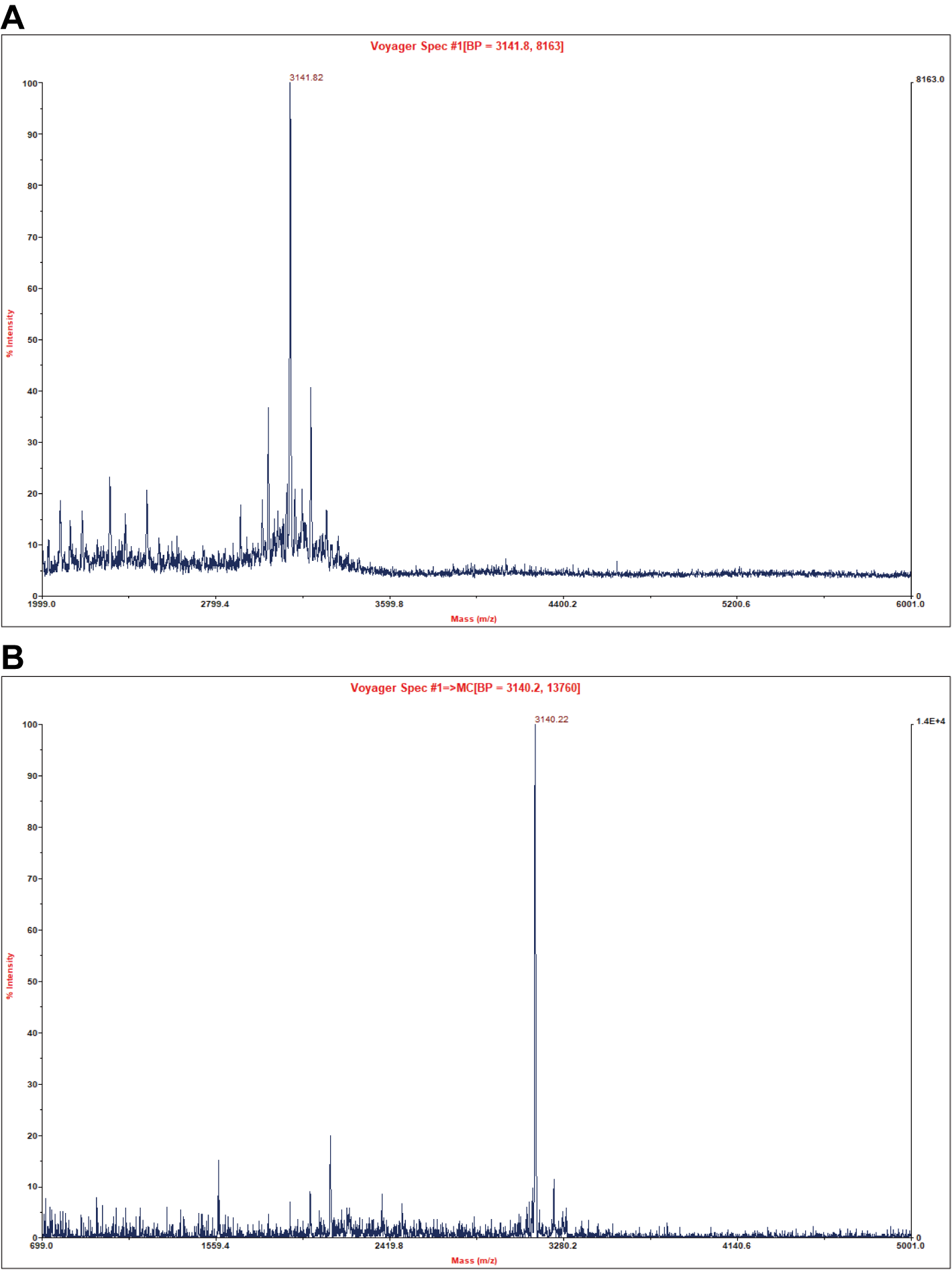

Supplement: Supplementary file 2 [file Image2.TIF]

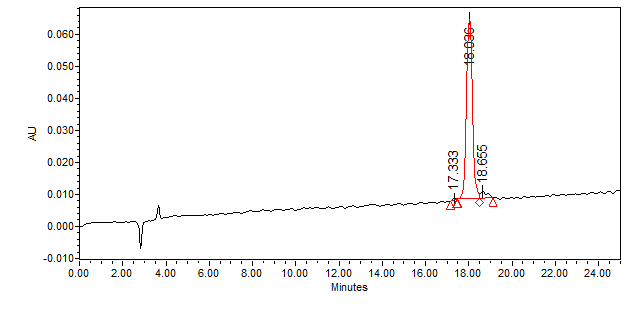

Supplement: Supplementary file 3 [file Image1.TIF]
